# Comparative virome analysis of lake and domestic wastewater revealed the unexpected presence of swine acute diarrhea syndrome coronavirus and *Ginkgo*-associated viruses

**DOI:** 10.3389/fmicb.2026.1831710

**Published:** 2026-06-05

**Authors:** Fei Xu, Huiling Qin, Fan Yu, Zhengyi Qiu, Duo Zhang, Nan Li, Pengpeng Xiao

**Affiliations:** 1 Wenzhou Key Laboratory for Virology and Immunology, Institute of Virology, Wenzhou University, Wenzhou, China; 2 College of Veterinary Medicine, Jilin University, Changchun, China

**Keywords:** domestic wastewater, ecosystem virome, lake, phylogenetic analysis, swine acute diarrhea syndrome coronavirus

## Abstract

**Introduction:**

Viruses in water bodies pose potential threats to human health and ecological environments. Therefore, exploring viral diversity in human-associated lakes and domestic wastewater is crucial.

**Methods:**

In this study, we performed comparative viromic analysis of lake and domestic wastewater samples collected from the same water system in Wenzhou, China.

**Results:**

A total of 13,708,121 sequencing reads were classified into 30 viral families, including viruses associated with animals, plants, and algae. Alpha diversity analysis showed that the lake exhibited higher viral richness and evenness than domestic wastewater. Notably, two ginkgo-associated viruses potentially infecting arthropods were detected in the lake: *Ginkgo biloba* picorna-like virus and *Ginkgo biloba* dicistrovirus. These clustered evolutionarily with arthropod-infecting viruses, suggesting a potential capacity to infect insects. Furthermore, Swine acute diarrhea syndrome coronavirus (SADS-CoV) sequences were detected in urban water environments and clustered with previously reported SADS-CoV strains. Evolutionarily, this SADS-CoV clade is closely related to the bat coronavirus HKU2, sharing the same *Rhinacovirus*.

**Discussion:**

These findings expand our understanding of the aquatic ecosystem virome.

## 1 Introduction

As a vital biological entity, viruses have a profound impact on regulating the structure of microbial communities, driving biogeochemical cycles, and regulating host evolution (Tian et al., 2024; Zhang et al., 2022). Recently, the emergence of viral metagenomics has provided a powerful tool to explore the vast viral diversity in aquatic ecosystems. However, aquatic environments are not isolated. Through rainwater runoff (Zhang J. et al., 2023), agricultural drainage (Xu et al., 2026), and wastewater discharge (Bivins et al., 2020), rivers and lakes continuously receive exogenous viruses from terrestrial, human, and animal sources. Aquatic ecosystems are reservoirs of viral genetic diversity.

Aquatic environments frequently receive viral inputs from surrounding human activities and terrestrial ecosystems. *Ginkgo biloba picorna-like virus* and *Ginkgo biloba dicistrovirus*, members of the order *Picornavirales*, have previously been reported only in terrestrial environments (Cheng et al., 2021), while their distribution and ecological significance in aquatic environments remain unknown. Concurrently, most human pathogens (e.g., adenoviruses and coronaviruses) have been widely used as wastewater indicator viruses due to their association with fecal-oral transmission (Cuevas-Ferrando et al., 2021; Du et al., 2022; Kumthip et al., 2023). *Swine acute diarrhea syndrome coronavirus* (SADS-CoV) is a highly pathogenic enteric coronavirus with significant economic impacts on the swine industry (Liu et al., 2024; Zhang et al., 2024). Although this virus has primarily been detected in agricultural settings such as swine farms, the possibility of its introduction into adjacent water bodies through agricultural runoff cannot be overlooked. However, the occurrence of SADS-CoV in natural water bodies, its environmental persistence, and potential public health risks remain inadequately studied to date.

To assess viral diversity and potential risks in the aquatic environment, this study selected two water systems: domestic wastewater, which is a series of complex pollutants generated by daily human activities, and lakes, which are typical freshwater bodies regulated by natural ecological processes. Our main research objectives were to: (1) compare the distribution and diversity of the viromes in the lake and domestic wastewater; (2) investigate the unexpected presence and potential origin of the SADS-CoV in an urban water environment sample. and (3) trace the cross-ecosystem transmission of arthropod-associated Ginkgo viruses. Ultimately, this study aims to broaden our understanding of aquatic RNA viruses from both epidemiological and ecological perspectives.

## 2 Materials and methods

### 2.1 Sample collection and pre-treatment

**Figure 1:**
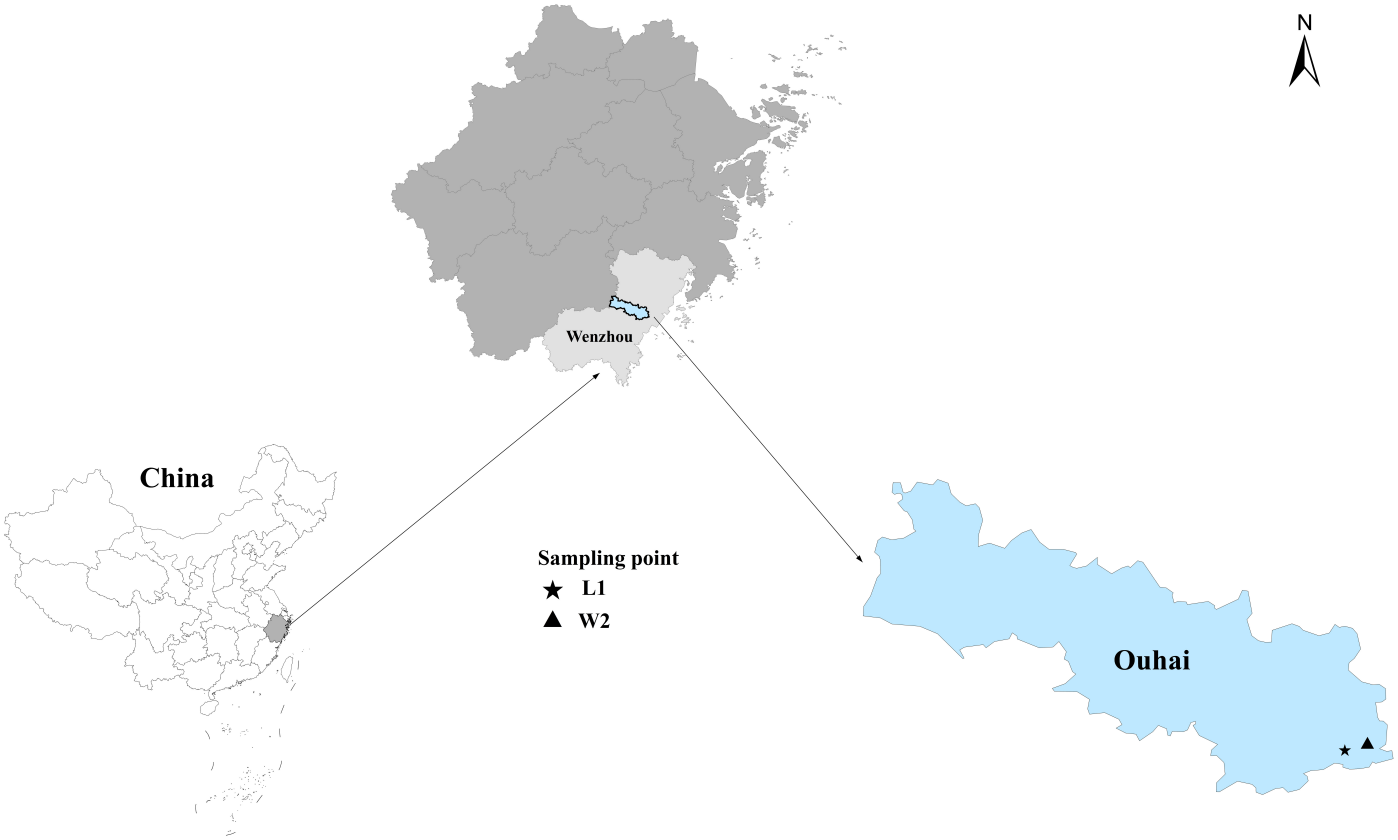
Map of the study area and sampling locations in Wenzhou, Zhejiang Province, China. L1: lake water sample collected from an artificial lake near areas of human activity. W2: domestic wastewater sample collected near a residential and commercial area.

Water samples (1,500 mL) were collected from two distinct sites in September 2024 in Ouhai District, Wenzhou, Zhejiang Province, China.The lake water sample (L1) was collected from an artificial lake with an approximate depth of 2–3 m. The domestic wastewater sample (W2) was collected from a flowing sewer outlet in a mixed residential and commercial area. Both sampling sites are adjacent to areas of frequent human activity (Figure 1). Both samples were transported at 4 °C to the laboratory and processed immediately. Specimens were concentrated as described previously (Tao et al., 2020). Briefly, 1,500 mL of each water sample was centrifuged at 6,000 × *g* for 20 min at 4 °C to remove large particles and debris. Subsequently, MgCl_2_ (2.5 M) was added to the supernatant to a final concentration of 0.05 M. The pH was adjusted to 3.5 with 0.5 M hydrochloric acid, imparting a positive charge to the viral particles and facilitating their electrostatic binding to the anionic membrane. These steps were performed on a magnetic stirrer. The solution was filtered through a mixed cellulose ester membrane filter with 0.45 μm (Jukai, Shandong, China) to maximize virus-membrane contact and capture efficiency. The membrane was removed in a biosafety cabinet, cut into small pieces, and placed in a 50 mL sterile centrifuge tube. To elute the adsorbed viruses, 15 mL of 3% beef extract solution was added to the membrane pieces, followed by ultrasonication for 20 min to facilitate the release of viral particles from the membrane. After centrifugation at 3,000 × *g* for 30 min at 4 °C, the supernatant was filtered through a 0.22 μm filter to remove residual bacteria. The pH of the filtrate was then adjusted back to 7.0 with hydrochloric acid to stabilize the viral particles (Elfellaki et al., 2024; Miura et al., 2021; Tao et al., 2020). Finally, the samples were stored at −80 °C and used for RNA extraction.

### 2.2 RNA extraction, library construction and sequencing

Total RNA was extracted from 1 mL of each concentrated viral sample using the HiPure Viral RNA kit (Magen, Guangzhou, China) following the manufacturer's instructions. The concentration of the extracted RNA was measured using NanoDrop2000 (ThermoFisher, USA) to determine the appropriate input amount for library construction. Extracted RNA was stored at −80 °C until further processing. Total RNA extracts were subjected to library construction using the VAHTS Universal V8 RNA-seq Library Prep Kit for Illumina (Vazyme, Nanjing, China). Ribosomal RNA (rRNA) of eukaryotic cytoplasm, mitochondria, and bacteria origin were removed using the Ribo-Clean rRNA Depletion Kit Mega (Bacteria) (Vazyme, Nanjing, China). The quantity and quality of the final RNA libraries were assessed using a Qubit 4.0 fluorometer (Invitrogen, USA). The RNA library construction was carried out in the laboratory, and the sequencing was performed by Beijing Novogene Technology Co., Ltd. Paired-end (150 bp reads) sequencing for each RNA library was performed on the Illumina HiSeq Xplus platform (Novogene, Beijing, China).

### 2.3 Bioinformatics analysis

After obtaining the raw data of each sample, the sequencing data (Raw data) were quality-controlled with FASTP (v.0.23.4) (Chen et al., 2018) to remove the adapters, low-quality and low-complexity reads, and to obtain high-quality clean reads to ensure the credibility of the results of the subsequent analysis. To remove host-derived reads, clean reads were first mapped against the Kraken 2 standard reference database using Bowtie 2 (v.2.4.2) (Wilton and Szalay, 2022). After host read removal, the remaining clean reads were taxonomically classified using Kraken 2 (v.2.0.9), and species-level abundance was estimated using Bracken (Lu et al., 2022a). The high-quality reads of each sample were then assembled into longer contigs using MEGAHIT (v.1.1.3) (Li et al., 2015), which was chosen for its high memory efficiency and suitability for the computational resources available in our laboratory.

### 2.4 Viral sequence identification and virus quantification

The assembled contigs were first aligned against the NCBI non-redundant protein database (nr) (https://www.ncbi.nlm.nih.gov) using DIAMOND blastx (https://github.com/bbuchfink/diamond) with an *E*-value threshold of < 1 × 10^−5^. Contigs annotated as viral were then further validated by aligning against the NCBI nucleotide sequence database (nt) using BLASTn with the same E-value threshold. Only contigs independently annotated as viral by both databases were retained as the final viral sequences, thereby minimizing false positives through cross-validation. Viral quantification was performed by mapping the clean reads back to the identified viral contigs and statistically analyzing the read counts.

### 2.5 Viral alpha diversity

Alpha diversity of the viral communities was assessed at both the family and genus levels. The Shannon index and Chao1 index were calculated using PAST (v.5.2.1) (Hammer et al., 2001). Visualization was generated via ChiPlot (https://www.chiplot.online/).

### 2.6 RT-PCR verification

Based on the results of contig comparison, RT-PCR was conducted to verify the assembled viral sequences and eliminate false positives. Viral contigs were assembled using SeqMan (v.7.1.0) from DNASTAR, and reference sequences were identified by aligning the assembled contigs against the NCBI nucleotide database using BLASTn. Primers were designed based on these reference sequences using Primer Premier 5 (See the Supplementary Table 1). Total RNA was extracted and purified using the HiPure Viral RNA Kit (Magen, Guangzhou, China) and reverse transcribed into cDNA. PCR amplification was performed under the following conditions: initial denaturation at 94 °C for 5 min; 35 cycles of 94 °C for 30 s, annealing at 50–60 °C for 30 s, and extension at 72 °C, with the extension time determined according to the size of each viral fragment; a final extension at 72 °C for 5 min. Negative controls were included in each run to rule out contamination. PCR products were visualized by agarose gel electrophoresis (Supplementary Figure 1). PCR products were sequenced by Sangon Biotech Co., Ltd. (Shanghai, China). The verified viral sequences were submitted to GenBank (Supplementary Table 2).

### 2.7 Phylogenetic analyses

Conserved protein-coding regions, including nucleoprotein (*N*) and other conserved hypothetical protein genes, were used for phylogenetic reconstruction in the respective viral taxa. The nucleotide sequences of the above viruses were subjected to sequence alignment using BLASTn against the NCBI nucleotide database., Reference sequences at the taxonomic family level were downloaded. Multiple sequence alignment was performed using MAFFT (v.7.520) with the E-INS-I algorithm (Katoh et al., 2019). After alignment, poorly aligned or low-quality regions were trimmed using TrimAl in TBtools (v.2.420) with the ML_AUTOMATED1 mode (Chen et al., 2023). After trimming, the phylogenetic analysis was conducted using the maximum likelihood method in IQ-TREE (v.2.4.0) (Nguyen et al., 2015) under the GTR+F+R3 model, with 1000 bootstrap replicates. The annotations and modifications were carried out using the online website iTOL (Letunic and Bork, 2021) (https://itol.embl.de/itol.cgi).

### 2.8 Genome annotation

Potential open reading frames (ORFs) were predicted using ORFfinder (https://www.ncbi.nlm.nih.gov/orffinder/with) the standard genetic code and a default minimum ORF length of 75 nt. The longest ORF in each sequence was selected for subsequent analysis. Conserved domains were searched against the InterPro database (https://www.ebi.ac.uk/interpro/search/sequence/), including Pfam and CDD. Signal peptides were predicted using SignalP 6.0 to identify potential secretory regions, and transmembrane helices were predicted using TMHMM 2.0 to identify membrane-spanning regions. Sequence profiles were further searched against the HHpred database (https://toolkit.tuebingen.mpg.de/tools/hhpred) to detect remote homologs. Amino acid conservation patterns among the viral sequences and their reference sequences were analyzed using Skylign (https://skylign.org/) to generate sequence logos and identify conserved residues. Multiple sequence alignments of the deduced amino acid sequences were performed using MEGAX (v.10.2.6). Mutation analysis, including the identification and visualization of amino acid substitutions relative to reference sequences, was conducted using BioAider (v.1.527) (Zhou et al., 2020), and the results were displayed as lollipop plots.

### 2.9 Host prediction

The Viral Host Predictor (http://host-predict.cvr.gla.ac.uk/) is used to predict the host of RNA viruses, arthropod vectors and arthropod-borne, including *Arenaviridae, Astroviridae, Bunyavirales* (*Feraviridae, Hantaviridae, Jonviridae, Nairoviridae, Peribunyaviridae, Phenuiviridae*, and *Tospoviridae*), *Caliciviridae, Coronaviridae, Filoviridae, Flaviviridae, Hepeviridae, Paramyxoviridae, Picornaviridae, Rhabdoviridae*, and *Togaviridae* (Babayan et al., 2018). At the same time, the RNAVirHost tool (Chen et al., 2024) is used to predict the host of the viral genomes. Visualization was generated via ChiPlot (https://www.chiplot.online/).

## 3 Results

### 3.1 Overview of metagenomic sequencing data

**Figure 2:**
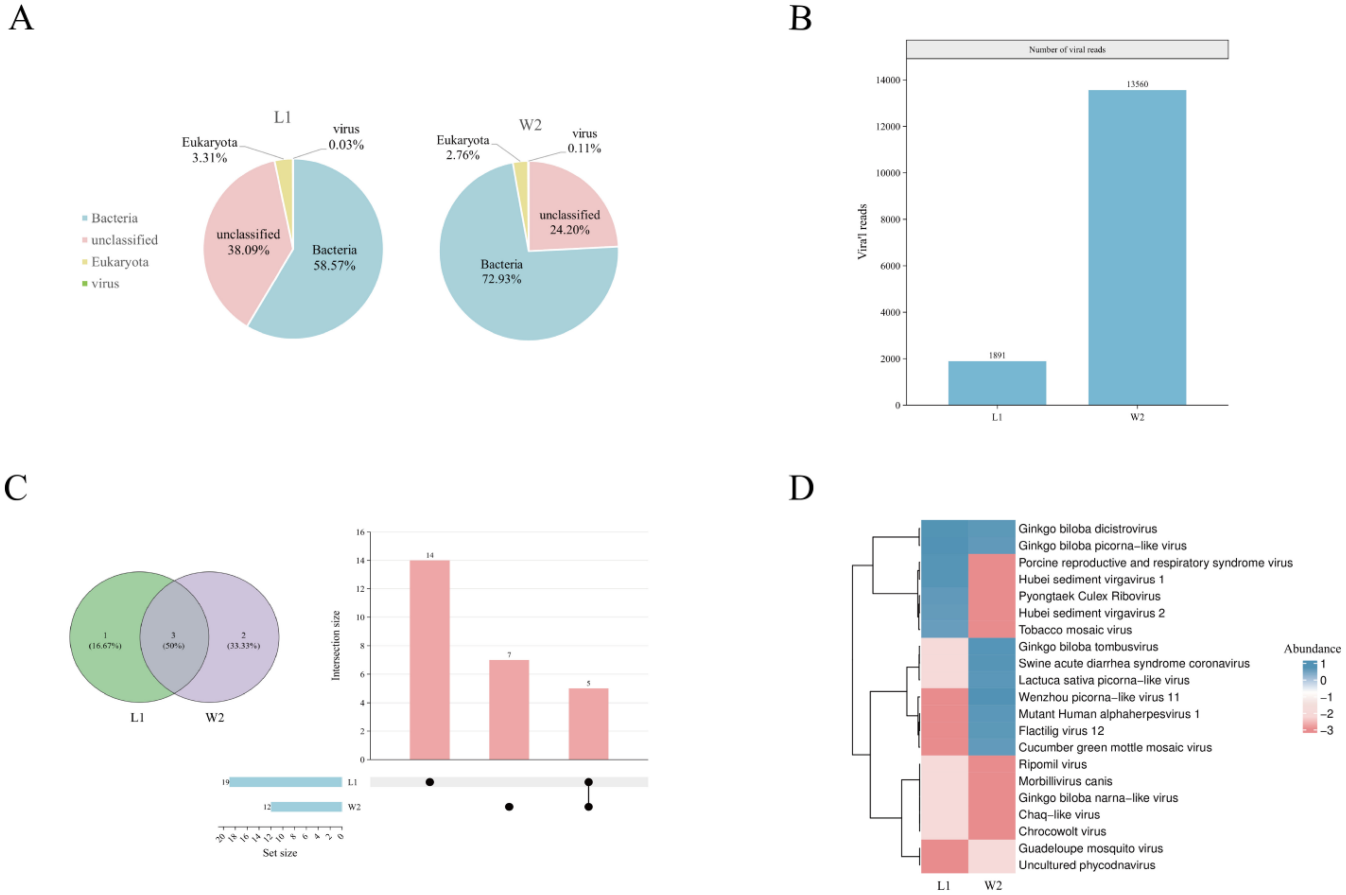
Overview of the two groups of virome samples. **(A)** The two groups of samples were annotated as bacteria, unclassified, eukaryotes, and viruses by Kraken2. **(B)** The two groups of samples were annotated with virus reads. **(C)** Intersection analysis of species type and virus differences between the two sample groups. **(D)** The abundance heatmap shows the abundance of viral contigs in the two groups of samples. The deeper the blue, the greater the abundance of the virus within the group.

After removing low-quality reads and host sequences, a total of clean bases is 4.6 Gb. Both groups were annotated as bacteria, eukaryotes, viruses and unknown sequences. The results indicated that bacteria remained an important component in water bodies. Although other biological categories were also identified, a large number of unknown sequences remain, indicating that many aspects of this field are still unknown and await further exploration (Table 1). In the virus category, the proportion of the lake of which 1891 were viruses, accounting for 0.03%, and that of the domestic wastewater libraries, of which 12560 were viruses, accounting for 0.11% (Figures 2A, B).

**Table 1:** Overview of sequencing libraries of different viromes.

Sample	Clean reads	Clean base (Gb)	Bacteria	Eukaryota	Unclassified	Virus	Viral read abundance (%)
L1	3740332	1.49	3260663	184305	2120248	1891	0.03
W2	9967789	3.17	9101450	344030	3019948	12560	0.1

The sequencing reads from the two sample groups were mainly annotated as bacteria, eukaryotes, viruses, and unclassified.

Virus discovery and identification were performed by assembling contigs from scratch, and species type and viral differences were statistically analyzed for the two sample groups. The results showed that a species type analysis of the two sample groups was performed using a Venn diagram. The two groups had three types in common: as plants, insects, and mammals. Among them, the lake (L1) had one unique species type, which belonged to the environmental sample, while the domestic wastewater (W2) had two unique types, as mollusc and Homo sapiens (Figure 2C). At the same time, we analyzed the viral species composition of two sets of libraries. The results showed that lake (L1) had the most viral species, with 19 species, while domestic wastewater (W2) had only 12 viral species. Among them, the lake (L1) carried 14 unique viruses, and the domestic wastewater (W2) carried 7 unique viruses. There were 5 shared viruses in the two libraries. This indicated that there were significant differences in the viral community composition of the two libraries (Figure 2C).

TPM is a standardized method for gene expression quantification, enabling accurate comparisons of expression levels within groups, between different groups, and between different viruses. The two groups of samples contained a total of 31 types of viruses. We calculated the relative abundance TPM of all viruses and through the abundance heatmap, which showed significant differences between the two libraries. In L1 library, the abundance of *Ginkgo biloba picorna-like virus* was the highest, accounting for 36.7%, followed by *Ginkgo biloba dicistrovirus* and *Porcine reproductive and respiratory syndrome virus*, accounting for 29.5% and 19.3%. In the W2 library, the *Wenzhou picorna-like virus 11* had the highest abundance, accounting for 45.6%, followed by *Ginkgo biloba* tombusvirus, accounting for 17.9% (Figure 2D).

### 3.2 Diversity of viral communities

**Figure 3:**
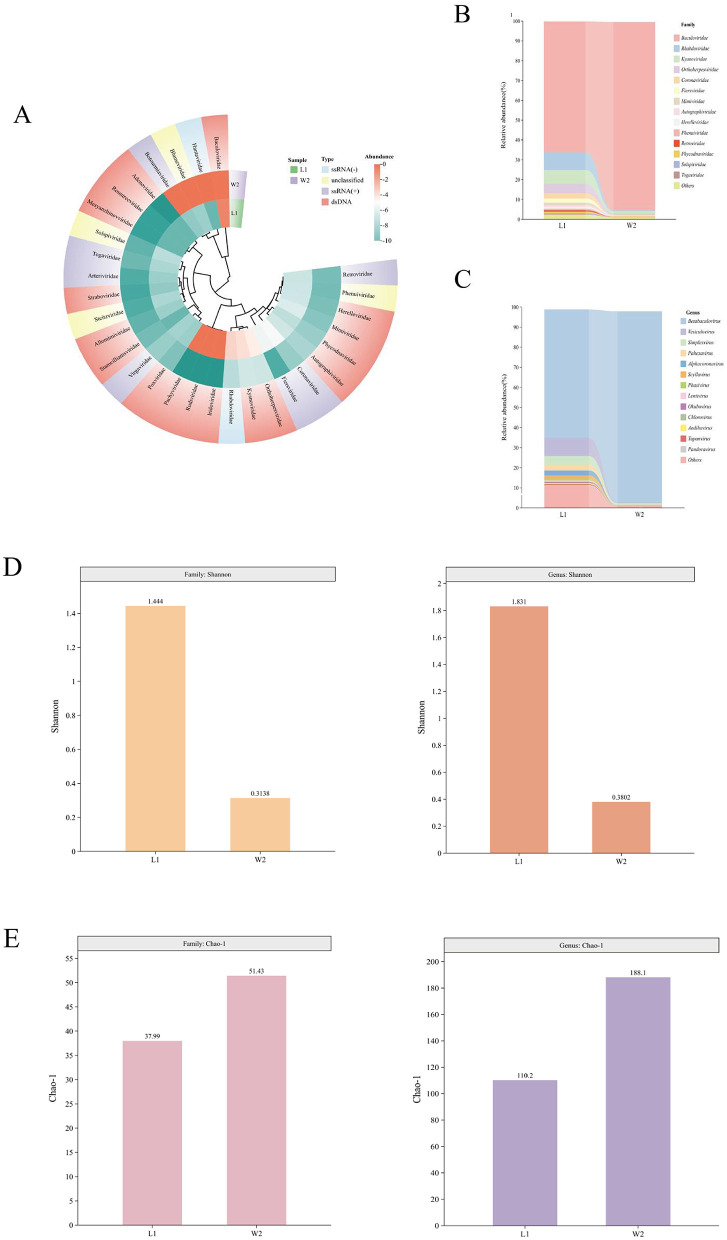
Statistical analysis of viral diversity. **(A)** Heat map showing the abundance of virus families in different RNA and DNA. The number of readings has been converted to log2. **(B)** The proportion of different virus families. **(C)** The proportion of different virus families. **(D)** Analysis of the Shannon index of alpha diversity at the viral family and genus level. **(E)** Analysis of the Chao-1 index of alpha diversity at the viral family and genus level.

To understand the distribution and abundance of viral families in lake and domestic wastewater, a heat map was constructed based on the viral genome sequences of the two libraries, and classified according to the family level. The viral reads were ultimately annotated to 30 viral families. In DNA viruses, *Baculoviridae, Adenoviridae, Rountreeviridae, Straboviridae, Poxviridae, Pachyviridae, Rudiviridae, Kyanoviridae, Orthoherpesviridae, Autographiviridae, Phycodnaviridae* and *Herelleviridae* had double-stranded DNA (dsDNA) genomes; In RNA viruses, *Hantaviridae, Paramyxoviridae* and *Rhabdoviridae* had single-stranded negative-sense RNA [ssRNA (-)] genomes; *Botourmiaviridae, Togaviridae, Arteriviridae, Virgaviridae, Fiersviridae* and *Coronaviridae* had single-stranded positive-sense RNA [ssRNA(+)] genomes; virus in the *Retroviridae* had single-stranded RNA reverse transcription (ssRNA-RT); *Blumeviridae, Solspiviridae, Steitzviridae* and *Phenuiviridae* were unclassified (Figure 3A).

At the specific families and genus level, *Baculoviridae*-*Betabaculovirus* had the highest proportion of all viruses, accounting for 66% in the lake and 95% in domestic wastewater. *Rhabdoviridae*-*Vesiculovirus* accounted for 8.99% in the lake and 0.5% in the domestic wastewater. It was clear that there were significant differences in the abundance of bacteriophages and plant viruses in the lake; in contrast, the lowest abundances were found in the domestic wastewater (Figures 3B, C).

Alpha diversity is key to understanding viral diversity and richness in sample libraries. The Chao-1 index can effectively estimate the number of species, while the Shannon index showed the extent of species diversity. The results analyzed the alpha diversity of the two libraries at the family and genus levels. At the family and genus levels, the Shannon index of L1 libraries is higher than W2 libraries. However, the number of W2 species was higher on the Chao-1 index (Figures 3D, E). The results showed that the species richness in the L1 libraries was relatively high, and the uniformity of community distribution was quite significant. In the W2 libraries, the viral diversity showed a relatively high species abundance. The community structure presented dominance by a few species, with a low degree of uniformity and a simple overall structure.

### 3.3 Phylogenetic analyses of viral sequences

#### 3.3.1 Virgaviridae

**Figure 4:**
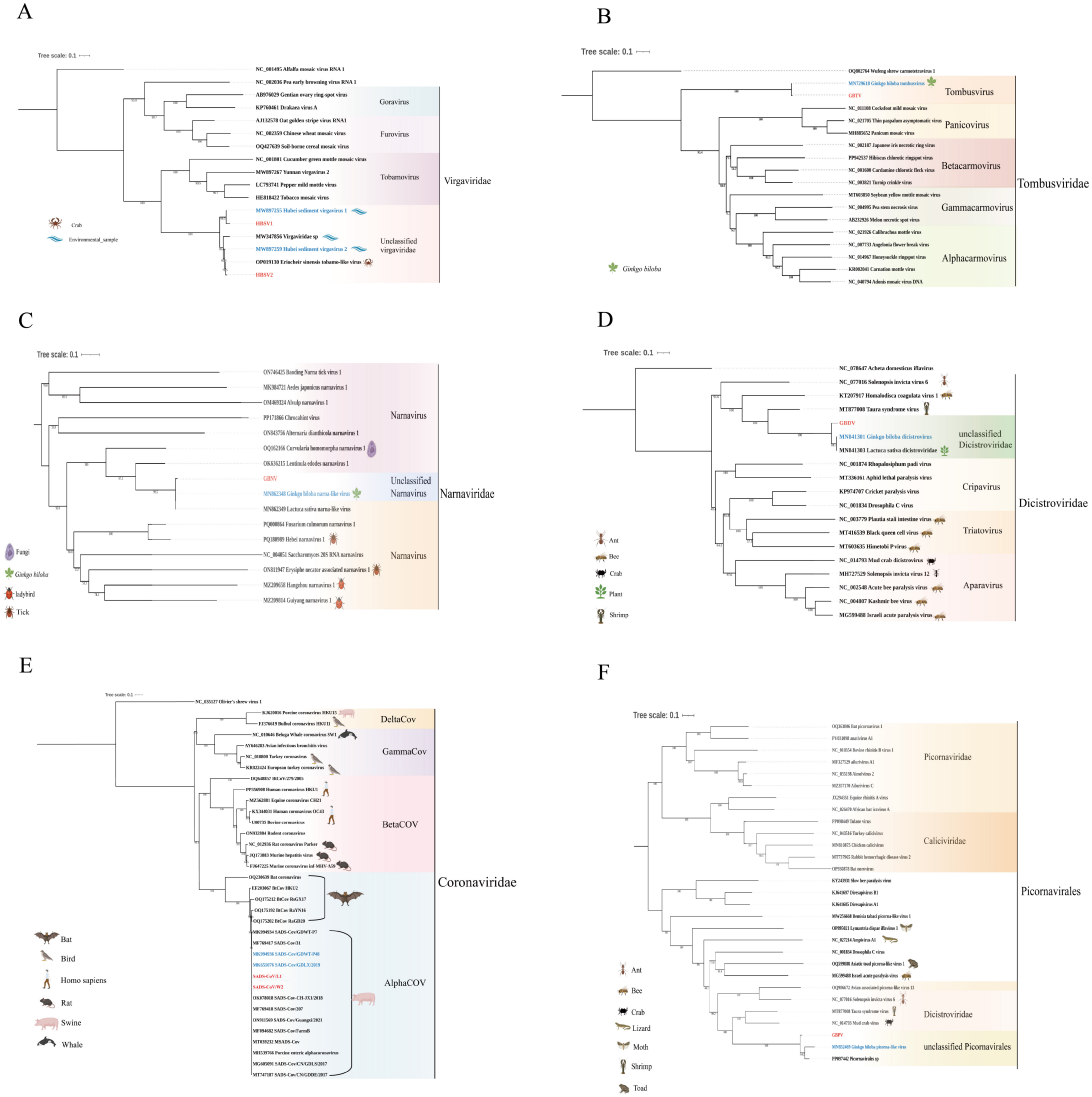
The genetic evolution analysis of viruses. **(A)** Partial hypothetical protein gene nucleotide sequences of Virgaviridae. **(B)** Hyp3 gene nucleotide sequence of Tombusviridae. **(C)** Partial gene nucleotide sequences of Narnaviridae. **(D)** Partial gene nucleotide sequences of Dicistroviridae. **(E)** N gene sequence of Coronaviridae. **(F)** Partial nucleotide sequences of hypothetical protein genes in Picornavirales.

Two viral sequences belonging to *Virgaviridae*, a family of plant-infecting single-stranded RNA viruses (Lu et al., 2024), were assembled from the lake sample (L1). *Hubei sediment virgavirus 1* (HBSV1) sequence showed nt identity of 95%−97% to the reference sequence MW897255, and *Hubei sediment virgavirus 2* (HBSV2) sequence showed nt identity of 96%−97% to the reference sequence MW897259, both of which were reported from Hubei, China in 2017.Phylogenetic analysis placed both sequences within the *unclassified Virgaviridae* clade (Figure 4A).

#### 3.3.2 Tombusviridae

A *Ginkgo biloba* tombusvirus (GBTV) sequence belonging to *Tombusviridae*, a family of plant-infecting viruses, was assembled from the lake sample. This sequence shared 99.4% nucleotide identity with a previously reported *Ginkgo biloba* tombusvirus (Genbank: MN729618) from Zhejiang, China in 2019. Phylogenetic analysis placedthis sequence withinthe *Tombusvirus* and was most closely related to MN729618.1 (Figure 4B).

#### 3.3.3 Narnaviridae

A *Ginkgo biloba* narna-like virus (GBNV) sequence showing similarity to *Narnavirus*, currently the only recognized genus within the *Narnaviridae* according to the latest ICTV classification, was identified in the lake sample and subsequently confirmed by RT-PCR. The result of the nucleotide sequence alignment showed that this sequence shared 98.8% identity with *Ginkgo biloba* narna-like virus (GenBank: MN862348) and *Lactuca sativa* narna-like virus (GenBank: MN862349), both previously reported from Jiangsu, China in 2019. Phylogenetic analysis showed that the three sequences are clustered on the same branch (Figure 4C).

#### 3.3.4 Dicistroviridae

A *Ginkgo biloba* dicistrovirus (GBDV) sequence, belonging to *Dicistroviridae*, was identified in the lake sample. Through RT-PCR and alignment, this sequence shared 91.88% nt identity with the reference sequence *Ginkgo biloba* dicistrovirus (GenBank: MN841301) from Jiangsu, China in 2019. Phylogenetic analysis showed that the sequences were clustered in the same branch, within the unclassified *Dicistroviridae* group (Figure 4D).

#### 3.3.5 Coronaviridae

Two N gene nucleotide sequences of SADS-CoV were assembled from the lake and domestic wastewater samples, respectively. SADS-CoV belongs to the genus *Alphacoronavirus* within the *Coronaviridae*. Among the four major structural proteins encoded by coronaviruses, the nucleocapsid (N) protein is highly conserved (Minigulov et al., 2024; Yan et al., 2022). Both sequences shared 100% nucleotide identity with MK994936 from Guangdong, China in 2019. Phylogenetic analysis placed both sequences within the *Alphacoronavirus*, clustering closely with known SADS-CoV strains (Figure 4E).

#### 3.3.6 Picornavirales

In this study, a nucleotide sequence (1388 nt) of *Ginkgo biloba* picorna-like virus (GBPV) was assembled from the lake library. GBPV belongs to the *Picornavirales*, a diverse group of non-enveloped positive-sense single-stranded RNA viruses that infect a broad range of eukaryotic hosts (Zell et al., 2024). This sequence shared 92%−94.9% nt identity with *Picornavirales sp*. (GenBank: PP097442) from Brazil and GBPV-pt112-upi-3 (GenBank: MN832469) from China. The phylogenetic analysis showed that the three sequences clustered on the same branch and belonged to the unclassified *Picornaviridae* (Figure 4F).

### 3.4 Virus host prediction

**Figure 5:**
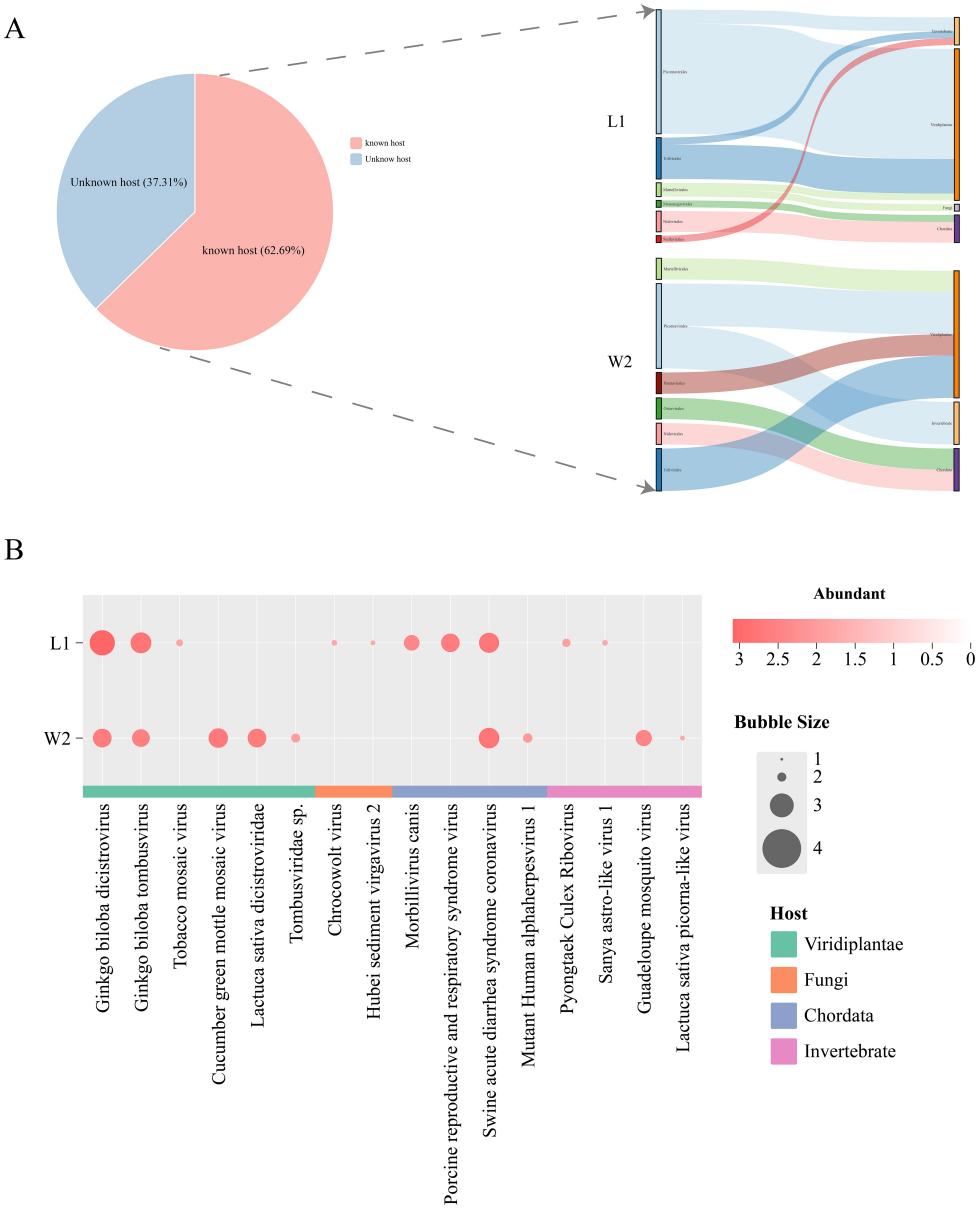
Virus-host association. **(A)** Virus-host linkage profiles showing the hierarchical classification of predicted hosts on the virus order. **(B)** The bubble plot showed the level of host classification predicted by the machine model. The deeper the red color, the greater the abundance.

To elucidate the host ranges of the identified virome, host prediction analysis was conducted. Overall, potential hosts were successfully predicted for 62.69% of the viral sequences, while 37.31% remained classified as having unknown hosts (Figure 5A). In lake (L1) and domestic wastewater (W2), the identified viral communities were predominantly predicted to infect Viridiplantae and Invertebrata (Figure 5A). Notably, viruses belonging to the order *Picornavirales* constituted a major proportion of the community. Further detailed analysis of specific viral species abundance across different host categories revealed distinct distribution patterns (Figure 5B). Viruses targeting Viridiplantae exhibited the highest relative abundance overall. Notably, the highly conserved *Ginkgo biloba* dicistrovirus and *Ginkgo biloba* tombusvirus were the most dominant species, showing exceptionally high abundance in both L1 and W2 samples. Most prominently, Swine acute diarrhea syndrome coronavirus displayed unexpectedly high relative abundance in both samples (Figure 5B).

### 3.5 Analysis of potentially harmful viruses

#### 3.5.1 Characteristics of the *Ginkgo* virus

**Figure 6:**
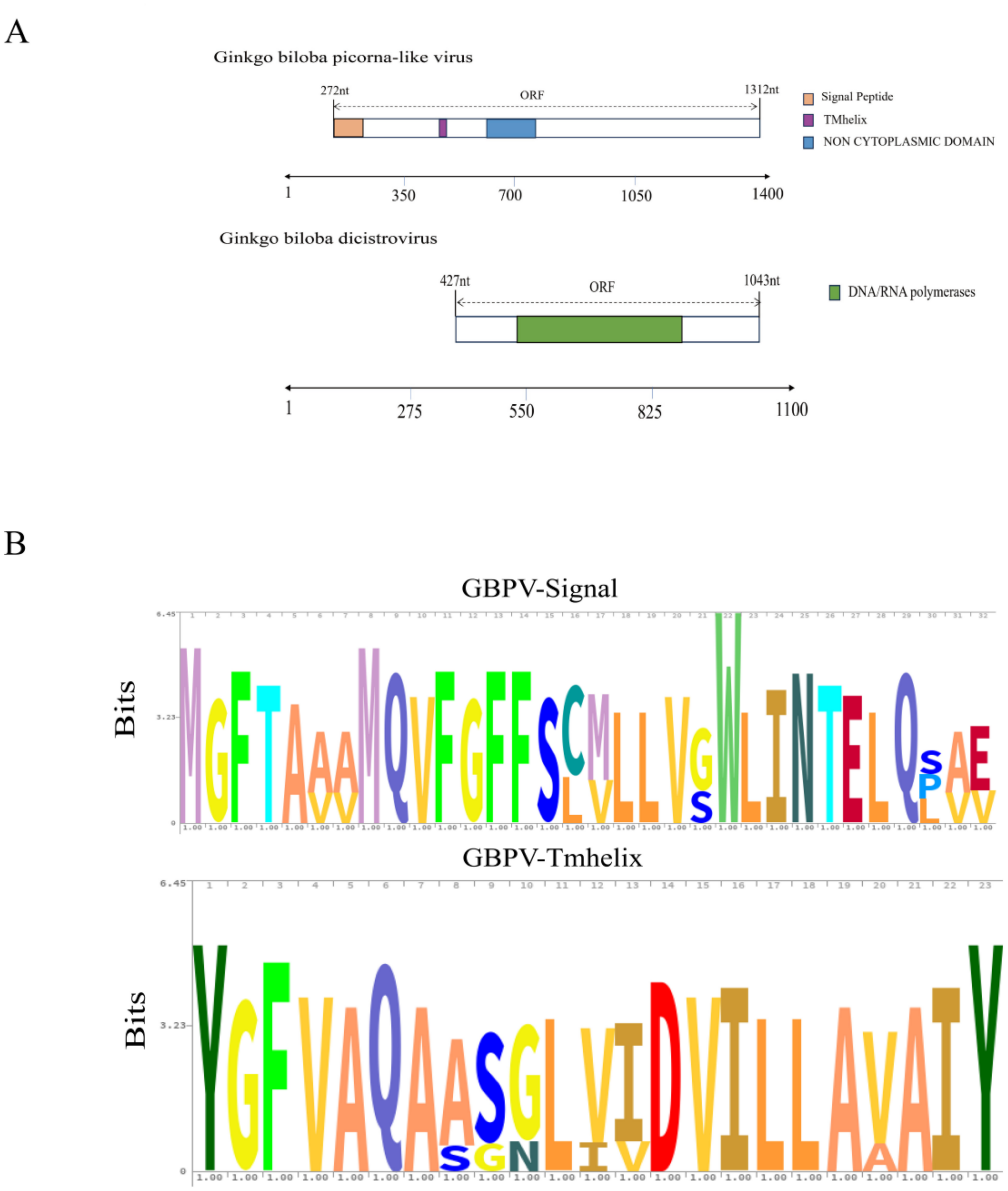
Analysis of the genomic structure and conservation of water samples. **(A)** The genomic structures of *Ginkgo biloba* picorna-like virus. The genomic structures of *Ginkgo biloba* dicistrovirus. **(B)** Conservation analysis of the signal peptide of *Ginkgo biloba* picorna-like virus. Conservation analysis of the transmembrane helix of *Ginkgo biloba* picorna-like virus. The X-axis represents the position of the amino acid, the Y-axis represents frequency, measured in probability. The higher the frequency, the more conservative the position.

*Picornavirales* members infect diverse hosts globally, driving major agricultural losses (Zhang Y. et al., 2023). In this study, two novel viral sequences from *Ginkgo biloba* were characterized: *Ginkgo biloba* picorna-like virus (GBPV) and *Ginkgo biloba* dicistrovirus (GBDV). GBPV consists of 1312 nucleotides (nt) containing nine ORFs (Supplementary Figure 2). The longest ORF (nt 272–1312) encodes a hypothetical protein sharing 85% amino acid identity and 97% coverage with an unclassified *Gingko biloba*-like virus (QKK82976) previously reported in China (Yang et al., 2022). Structural predictions revealed that this protein possesses a Sec/SPI-type signal peptide (SP), a transmembrane (TM) helix, and a non-cytoplasmic domain (Figure 6A), strongly suggesting it is a type I transmembrane or secretory protein (Supplementary Figure 3). Specifically, the SP cleavage site is predicted between amino acids 33 and 34 (probability: 0.989).

Further conservation analysis of the GBPV SP and TM domains highlighted a high frequency of hydrophobic residues, such as M and W at positions 1 and 21 in the SP, and Y in the TM domain (Figure 6B), indicating highly conserved hydrophobic functions. The SP sequence exhibited nine mutations (e.g., C16L and C33V), while the TM domain contained ten variations, including N203D/E and A212S (Supplementary Figure 4). These substitutions could potentially alter the protein's structural conformation and biological characteristics.

For GBDV (1044 nt), the single predicted ORF (nt 426-1043) encodes a putative DNA/RNA polymerase (aa 537-857, IPR043502) (Figure 6A). This sequence shares 97.57% amino acid identity and 100% coverage with a *Lactuca sativa* dicistroviridae (QKI28881). To further elucidate its structure, HHpred analysis was performed. The GBDV protein showed the highest structural homology to the known 6R1I and 8C1N_A proteins, with structural prediction probabilities reaching 99.88% and 99.87%, respectively (Supplementary Figure 5).

#### 3.5.2 Potential host

**Figure 7:**
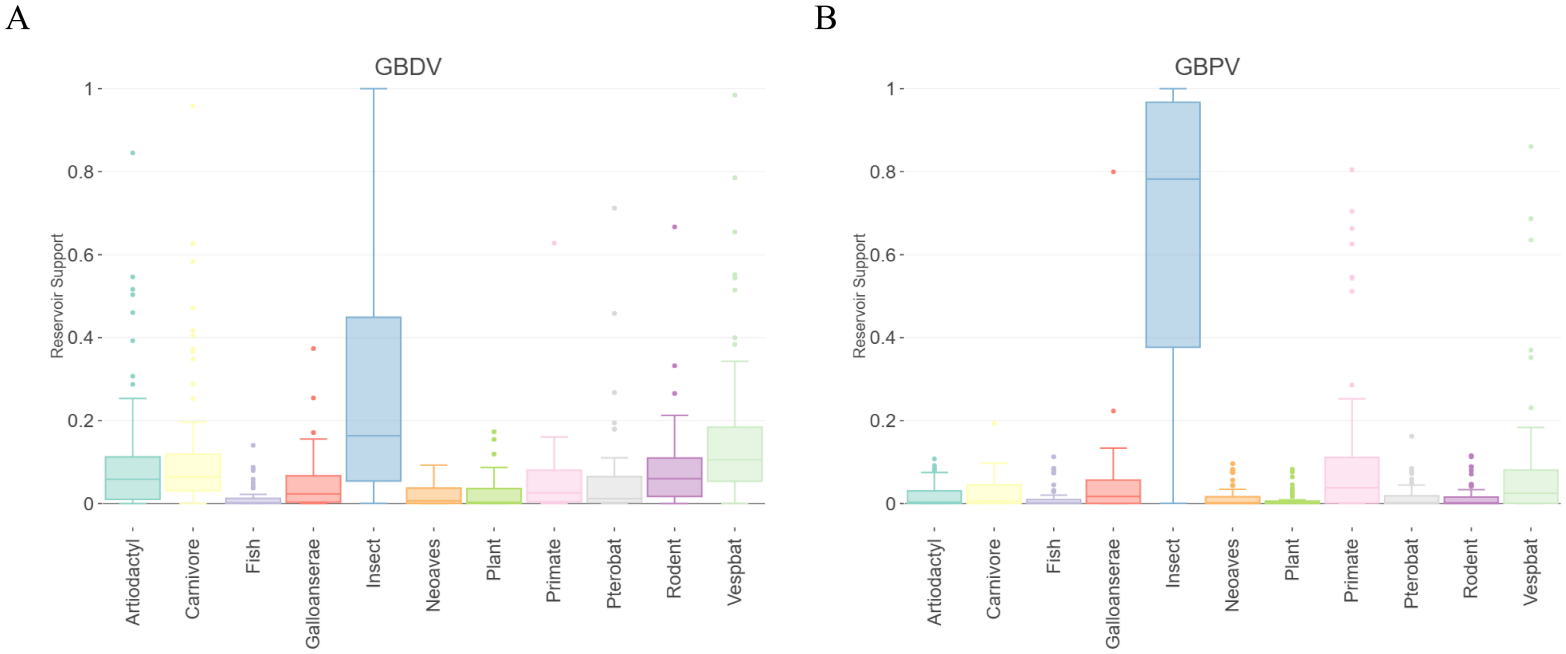
Host predictions were made for *Ginkgo biloba* dicistrovirus and *Ginkgo biloba* picorna-like virus in the L1 group library. **(A)** Prediction of the host for the *Ginkgo biloba* dicistrovirus. **(B)** Prediction of the host for the *Ginkgo biloba* picorna-like virus. The x-axis represents the predicted host names in the analysis. The y-axis represents the probability of statistical test support. The box plot is used to display the average value and statistical dispersion of the values obtained during the host prediction process.

In this study, by host prediction models based on genomic biases and phylogenetic neighborhood, the results showed that among the potential hosts of *Ginkgo biloba* dicistrovirus (GBDV, PV874581). *Ginkgo biloba* picorna-like virus (GBPV, PV925707), insects had a higher probability of matching than other host groups (such as plants, primate), indicating that both viruses are more likely to infect insect hosts (Figures 7A, B). In addition, phylogenetic analysis of GBDV and GBPV showed that the viruses infecting arthropods clustered in the same branch of the phylogenetic tree with high phylogenetic affinity (100%) (Supplementary Figure 6).

#### 3.5.3 Characterization of swine acute diarrhea syndrome coronavirus

**Figure 8:**
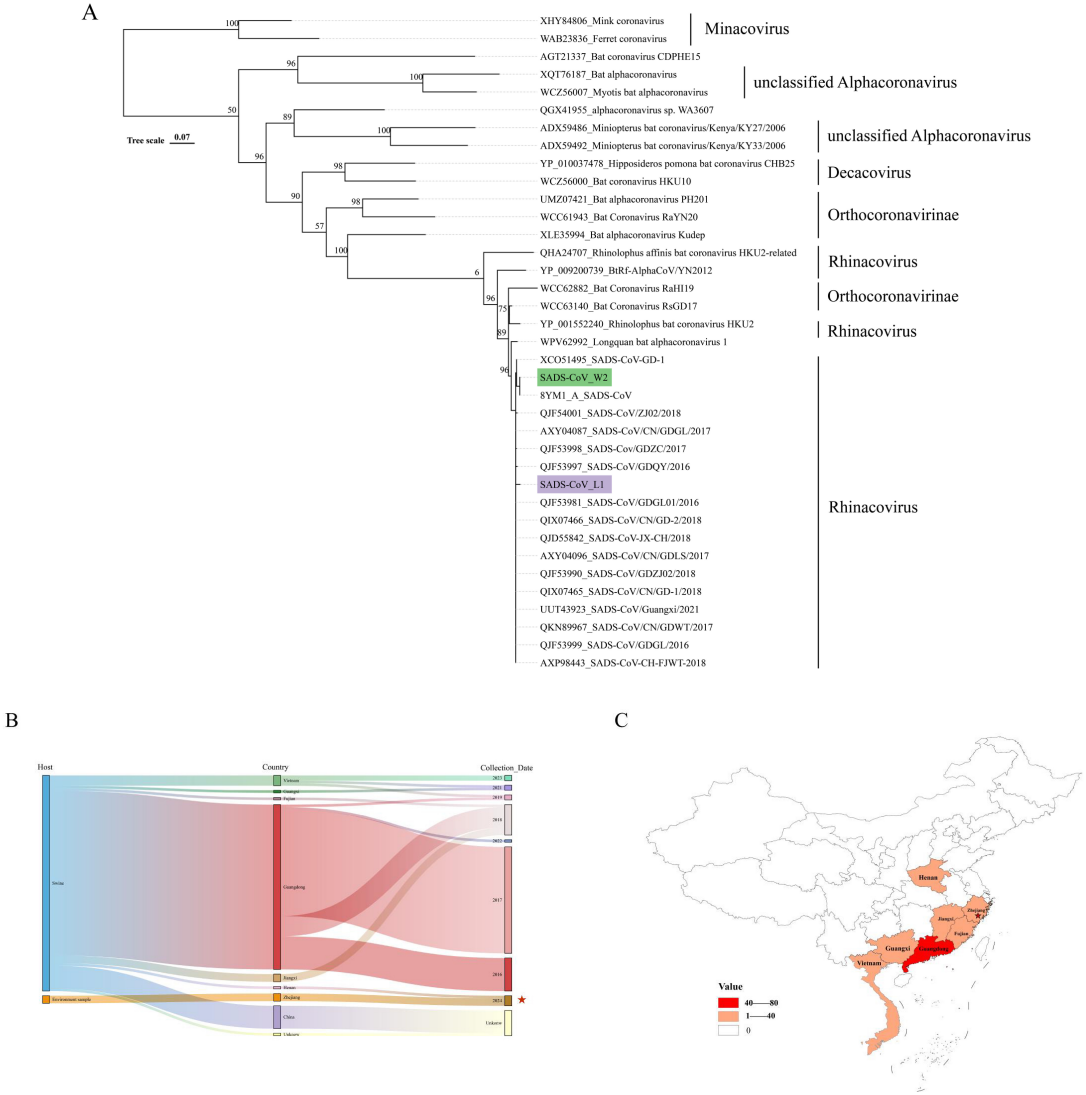
The distribution characteristics of Swine acute diarrhea syndrome coronavirus (SADS-CoV). **(A)** Phylogenetic analysis of SADS-CoV N genes detected in Zhejiang, China. The green indicate domestic wastewater (W2) samples. The purple indicate domestic lake (L1) samples. **(B)** The distribution of the N gene of all SADS-CoV strains on NCBI database. **(C)** The map showing the current country distribution of SADS-CoV N genes and statistics on the number of sequences.

We successfully identified swine acute diarrhea syndrome coronavirus (SADS-CoV) sequences in both groups using RT-PCR. A phylogenetic tree was constructed based on the N protein gene predicted from the open reading frame (ORF). Phylogenetic analysis indicated that the two sequences were closely related to SADS-CoV strains circulating in China between 2017 and 2018, showing 98.68% amino acid identify with the 8Y8M_A strain. This SADS-CoV clade clusters closely with bat coronavirus HKU2, and both are members of the *Rhinacovirus* subgenus (Figure 8A). By March 2026, a comprehensive metadata analysis was conducted on all available SADS-CoV nucleocapsid protein (N) sequences retrieved from the NCBI database. Among the historical host reservoir of SADS-CoV, swine hosts constitute the overwhelming majority. In terms of geographical distribution, the period from 2016 to 2018 saw a high incidence of SADS-CoV outbreaks in Guangdong Province, China (Figures 8B, C). Notably, our detection introduces a rare sequence originating from an environment sample rather than a direct swine host. This finding not only expands the recognized host categories in public databases but also provides critical evidence for the silent persistence or recent undetected circulation of SADS-CoV in environmental samples.

## 4 Discussion

Previously, metagenomic studies of Wenzhou water bodies have revealed the interaction between microorganisms and antibiotic resistance genes, which greatly enriches our understanding of the water environment in Wenzhou (Ye et al., 2023). Our research will further analyze the diversity of unknown viromes in the water environment, reveal the impact of virus distribution and diversity, and monitor the potential viral risks in the water environment.

In this study, we found a highly diverse virome in the aquatic environment, including viruses that infect algal, plants, and animals. The bacteriophages of *Rudiviridae* and *Fiersviridae* in the lake and *Solspiviridae, Kyanoviridae*, and *Blumeviridae* in wastewater were dominant, and this numerical advantage was similar to the research findings of water ecosystems such as the Yangtze River (Lu et al., 2022b), rivers (Hussain et al., 2025) and lake (Butina et al., 2021). A few algal virus families may regulate the population structure of aquatic zooplankton (Biggs et al., 2021).

At the same time, we detected the *Adenoviridae* and the *Coronaviridae* in both groups of water samples. The *Adenoviridae* is an established indicator of fecal contamination and an important waterborne pathogen causing human diseases (Owliaee et al., 2024; Pedrosa de Macena et al., 2023). A major and unexpected finding is the detection of the SADS-CoV in both lake and domestic wastewater samples. To our knowledge, this study reports the detection of SADS-CoV-like sequences in an environmental water sample, as previous studies have strictly isolated this virus from pig farms (Zhou et al., 2018). Interestingly, our sampling sites were located near residential areas and food stalls, so it is possible that the SADS-CoV-like detected in our samples did not originate from live pigs, but more likely from domestic sewage and kitchen waste. Food stalls and residents frequently wash raw pork products. If these pork products are contaminated with the virus, the wash water and food residues can easily introduce viral fragments into the local drainage system. Furthermore, during heavy rainfall, untreated wastewater enters lakes via storm drains, thereby entering the same water system. Previous study suggested that pig feces or feces from other mammals may excrete a large number of viruses, which enter the water environment through runoff or wastewater discharge, which is consistent with the known fecal-oral transmission route (Liu et al., 2024). Some studies have also shown that related coronaviruses can persist under low temperature conditions (Mohan et al., 2021). This study provides a basis for early monitoring and preventing the spread of coronavirus in low-temperature water environments.

Although the detection of SADS-CoV-like sequences is noteworthy, the short fragments recovered may be related to the recovery efficiency of the concentration method employed, which warrants careful consideration. The anionic membrane adsorption method relies on electrostatic interactions between viral particles and the negatively charged membrane. Non-enveloped viruses possess a stable protein capsid that is relatively resistant to pH changes and physical forces during concentration, and may therefore be recovered at higher efficiencies. In contrast, enveloped viruses including SADS-CoV have a lipid bilayer that is more vulnerable to the chemical and physical conditions encountered during processing, which may reduce their recovery. The differential recovery of enveloped and non-enveloped viruses has also been observed in previous comparisons of virus concentration methods (Ahmed et al., 2020; Farkas et al., 2022). Consequently, this may have influenced the observed proportions of different virus types in our virome data. Future studies employing complementary concentration techniques would help address this bias.

*Baculoviridae* mainly use insects as hosts (Rodríguez-Hernández et al., 2023). In the research results, the relative abundance of the *Baculoviridae*-*Baculovirus* was the highest in both groups of water samples (reaching over 65%), indicating that baculoviruses can continue to exist in an environment without insect hosts, and can stably exist in surface water and sediments for weeks to months (López-Ferber et al., 2025; Williams et al., 2017).

In addition to the animal virus, we also discovered two specific viruses: *Ginkgo biloba* picorna-like virus (GBPV) and *Ginkgo biloba* dicistrovirus (GBDV). These viruses were originally discovered in terrestrial *Ginkgo* plant leaves (Yang et al., 2022), and our results showed that both viruses belong to the *Picornavirales* through virome and phylogenetic analysis. The viral sequence and phylogenetic analysis of *Ginkgo biloba* dicistrovirus belonged to the *Dicistroviridae*, whose members are almost all insect viruses. The robust, non-enveloped capsid of *Dicistroviridae* suggested that they are stable in the environment for long periods, allowing them to be transported and detected (Cheng et al., 2021). Meanwhile, the phylogenetic analysis showed that the two viruses clustered in the same branch with some arthropods. Secondly, the host prediction results also emphasized this conclusion. It showed that arthropods were the most likely hosts of the two viruses. *Ginkgo* trees may have served as passive virus reservoirs during the feeding activities of some arthropods. The detection of these viruses in the aquatic environment may be since rainstorms washing the virus particles from the source of urban trees into the water.

Monitoring of the aquatic viral community provides an important perspective for assessing viral contamination in lake and wastewater. Non-etheless, our study still had limitations. First, our sample size is relatively small, which may not fully represent the viral diversity of the whole region. Consequently, the comparative findings presented here should be viewed as preliminary observations. A larger-scale study with additional samples from multiple sites or time points might reveal greater viral diversity and could potentially lead to different comparative outcomes, particularly regarding the relative abundance and presence of specific viral taxa. Second, the viral sequences successfully validated in this study do not confirm the presence of infectious viral particles. Viral viability and infectivity were not assessed in this study. Future studies incorporating cell-culture-based infectivity assays are needed to evaluate whether the viruses detected in these environmental samples have pathogenic potential.Future work should: (i)expand the sample size and sampling scope to further validate our observations; (ii) include negative and process controls in the experimental workflow to distinguish genuine viral signals from potential contamination. (iii) employ deeper sequencing, targeted enrichment, or long-read sequencing technologies to obtain more complete viral genomes. (iv) incorporate cell-culture-based infectivity assays to assess whether the detected viruses have pathogenic potential.

## 5 Conclusions

In summary, our study showed that aquatic viral communities were complex assemblages influenced by both autochthonous and terrestrial inputs. The eventual detection of *Ginkgo biloba* picorna-like virus and *Ginkgo biloba* dicistrovirus highlighted the role of hydrological pathways in the transmission of viral genetic material across ecosystem boundaries. Furthermore, our investigation contributes to characterizing the environmental occurrence and potential public health implications of known coronavirus viruses (e.g., SADS-CoV) in waters environmental. This study expanded our knowledge of the aquatic virosphere and refreshed the understanding of the correlation between viruses in terrestrial and aquatic ecosystems.

## Data Availability

The datasets presented in this study can be found in online repositories. The names of the repository/repositories and accession number(s) can be found in the article/Supplementary material.
