# Supplementary material for: Comparative virome analysis of lake and domestic wastewater revealed the unexpected presence of swine acute diarrhea syndrome coronavirus and Ginkgo-associated viruses
Source: Front Microbiol. 2026 Jun 5;17:1831710. doi: 10.3389/fmicb.2026.1831710 (PMC13279605; doi:10.3389/fmicb.2026.1831710)
Supplement: Supplementary file 1 [file Supplementary_file_1.docx]

Supplementary Material

# Supplementary Tables

Table S1 Primers used for RT-PCR verification

| Virus (abbreviation) | Virus isolation source | Sequence (5′-3′) | Product Size (bp) | Tm (℃) |
| --- | --- | --- | --- | --- |
| Hubei sediment virgavirus 1 (HBSV1) | L1 libraries | F: CCACGATGGTTGTTGAAGTCCT  R: TGGGCACTAAAATCCATTCCTG | 459 | 59 |
| Hubei sediment virgavirus 2 (HBSV2) | L1 libraries | F: GATGCGAGTTTCTCTGATGGACA  R: ACTCTCTTTGTAGGTCCTTGCTGG | 273 | 59 |
| Ginkgo biloba tombusvirus (GBTV) | L1 libraries | F: CGGAGTATGTTGGGCAGTAATGT  R: CGGGCTTGGGTTCAATCTGT | 238 | 60 |
| Ginkgo biloba narna-like virus (GBNV) | L1 libraries | F: GGAAGATGTGGTTAAGCGTTGTG  R: CCGTTCTCAGCATCTGGGTTC | 249 | 59 |
| Ginkgo biloba dicistrovirus (GBDV) | L1 libraries | F: AGGTGTCAGTCCGAAGAACGAT  R: GGTCTCTCTCCTCTCAGAATCATCC | 1050 | 58 |
| Swine acute diarrhea syndrome coronavirus  (SADS-CoV) | L1 libraries | F: CGGCTCGTAAGCAGATGGAC  R: CTTGTGTCGGAAGCAACTCAGC | 177 | 60 |
| Ginkgo biloba picorna-like virus (GBPV)  Swine acute diarrhea syndrome coronavirus  (SADS-CoV) | L1 libraries  W2 libraries | F: CGGAGTATGTTGGGCAGTAATGT  R: CGGGCTTGGGTTCAATCTGT  F: CGGCTCGTAAGCAGATGGAC  R: CTTGTGTCGGAAGCAACTCAGC | 1412  214 | 58  59 |

Supplementary Tables 1. All primers were designed based on the assembled contigs obtained in this study. F, forward primer; R, reverse primer; Tm, annealing temperature.

Table S2 Viruses successfully identified by RT-PCR in the phylogenetic tree

| Virus (abbreviation) | Virus isolation source | Asscession |
| --- | --- | --- |
| Ginkgo biloba dicistrovirus（GBDV) | L1 libraries | PV874581 |
| Ginkgo biloba tombusvirus(GBTV) | L1 libraries | PV890914 |
| Ginkgo biloba narna-like virus(GBNV) | L1 libraries | PV890915 |
| Ginkgo biloba picorna-like virus GBPV) | L1 libraries | PV925707 |
| Swine acute diarrhea syndrome coronavirus（SADS-CoV) | L1 libraries | PV927310 |
| Swine acute diarrhea syndrome coronavirus（SADS-CoV) | W2 libraries | PV927311 |
| Hubei sediment virgavirus 1 (HBSV1) | L1 libraries | PV932130 |
| Hubei sediment virgavirus 2 (HBSV2) | L1 libraries | PV932131 |

# Supplementary Tables 2. Sequences successfully validated by RT-PCR in both sample groups. GenBank accession numbers are provided for all the sequences.

## Supplementary Figures

Figure S1


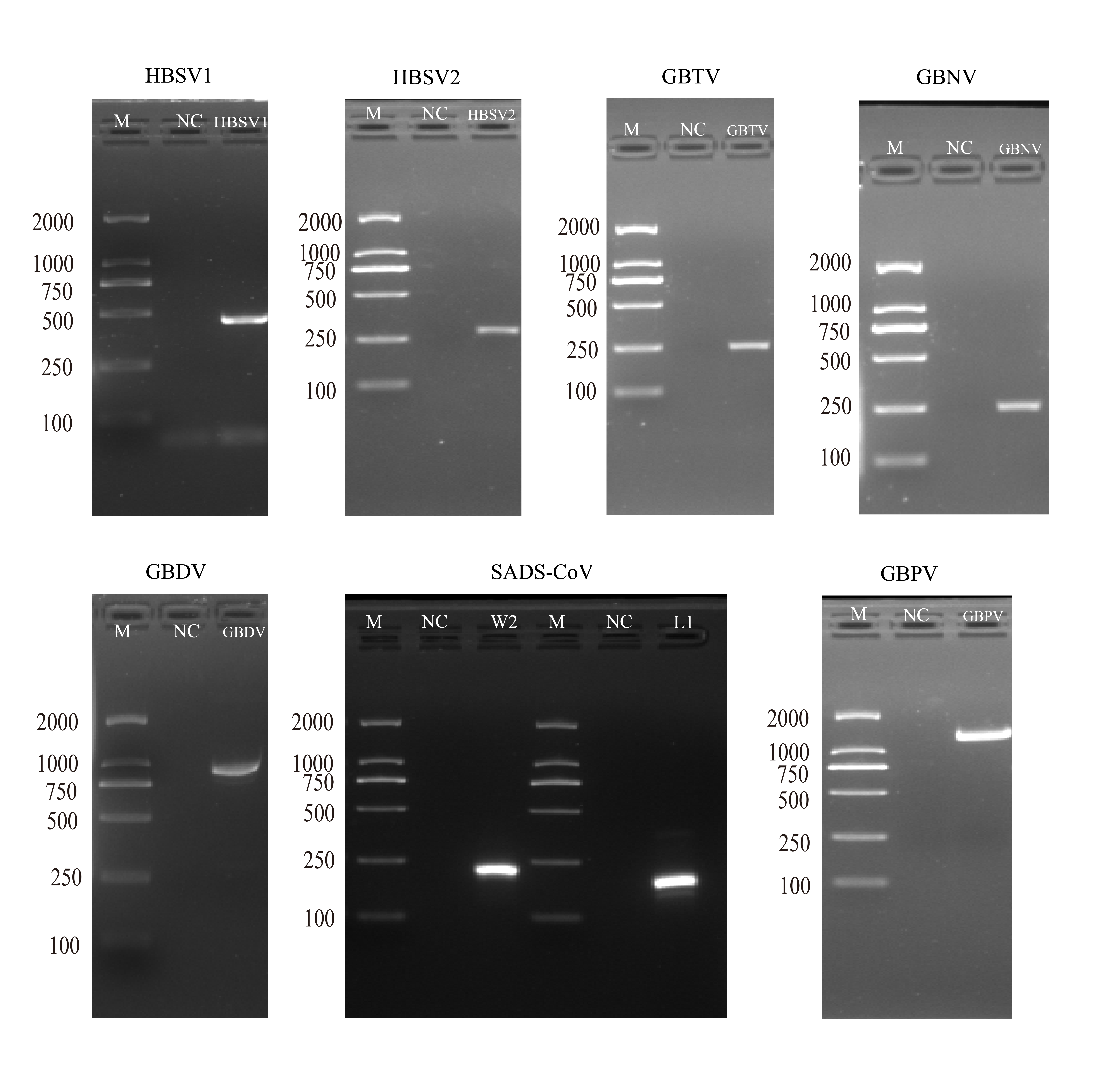


Supplementary Figure 1. RT-PCR validation of the identified viral sequences by agarose gel electrophoresis. M, DNA marker; NC, negative control. Hubei sediment virgavirus 1 (HBSV1): 459 bp; Hubei sediment virgavirus 2 (HBSV2):273bp; Ginkgo biloba tombusvirus (GBTV):238 bp; Ginkgo biloba narna-like virus (GBNV):249bp; Ginkgo biloba dicistrovirus (GBDV):1050 bp; Swine acute diarrhea syndrome coronavirus（SADS-CoV)(W2 libraries): 214 bp; (SADS-CoV) (L1 libraries): 177 bp; Ginkgo biloba picorna-like virus (GBPV): 1412. No amplification was observed in the negative control lanes.

Figure S2


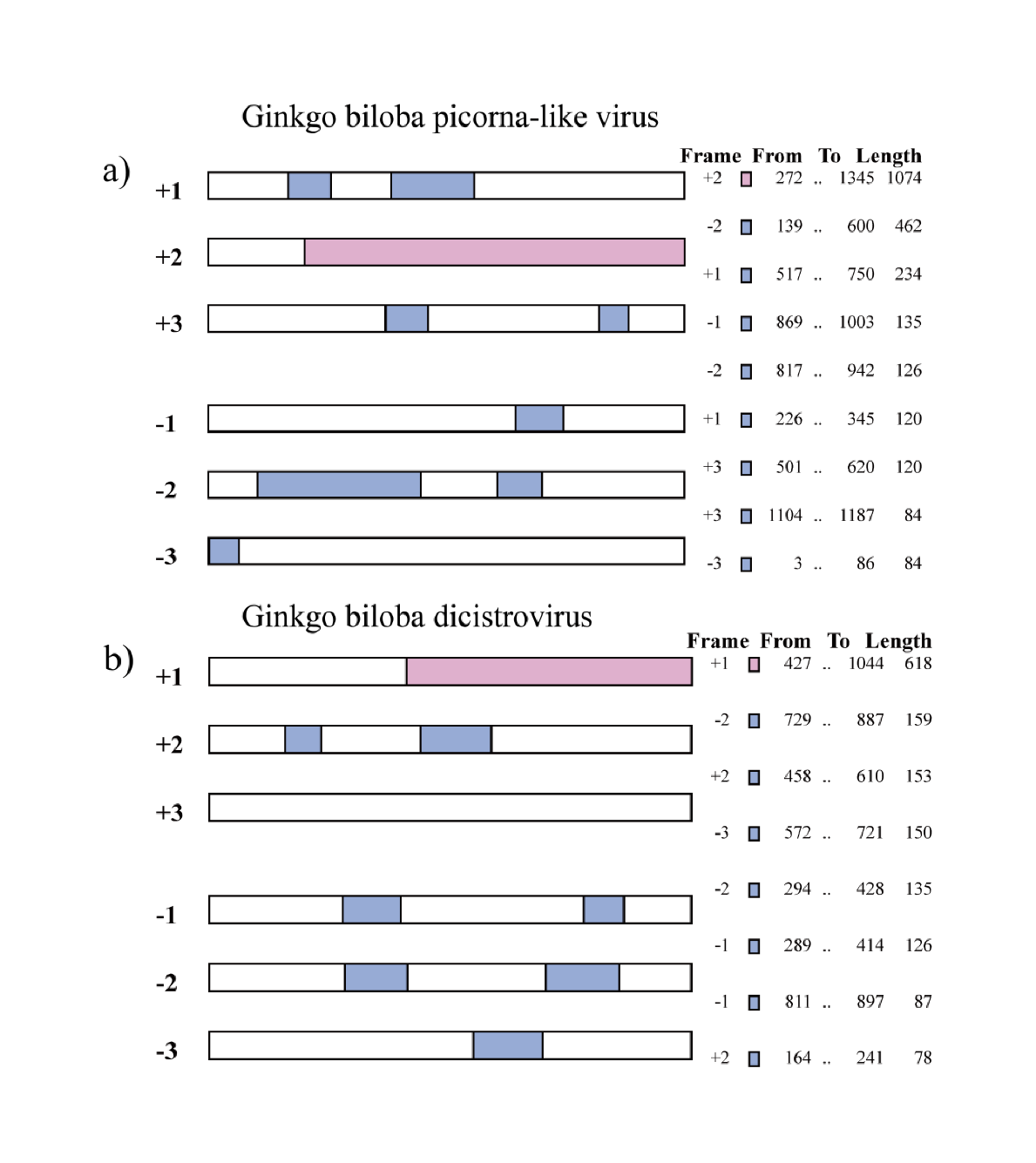


**Supplementary Figure 2.** The results of the open reading frames (ORF) of *Ginkgo biloba* picorna-like virus and *Ginkgo biloba* dicistrovirus. a) The results of the 6 ORFs of *Ginkgo biloba* picorna-like virus. b) The results of the 6 ORFs of *Ginkgo biloba* dicistrovirus. Pink shows the longest open reading frame.

Figure S3


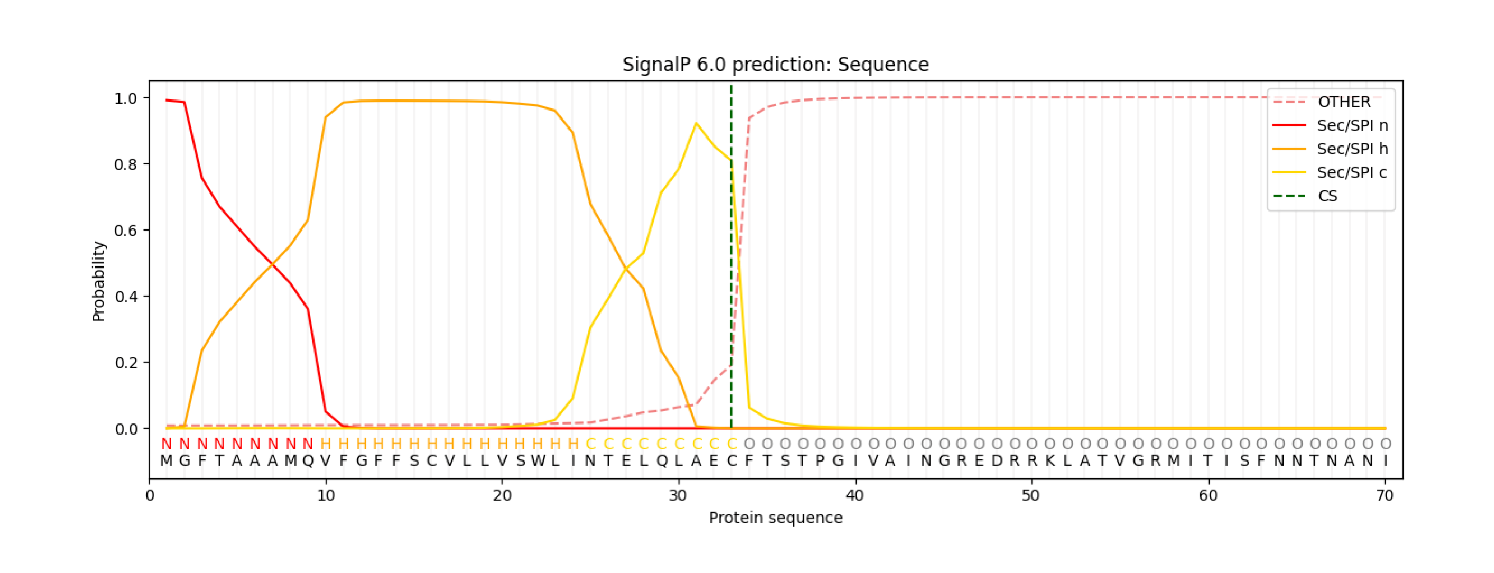


**Supplementary Figure 3.** The signal peptide found in the *Ginkgo biloba* picorna-like virus. The amino acids before the green line represent the predicted signal peptide region. N: Amino acids 1-9, the positively-charged signal region at the N-terminus. H: Amino acids 10-24, Central drainage core section. C: Amino acids 25-33, the C-terminal region close to the cleavage site.

Figure S4


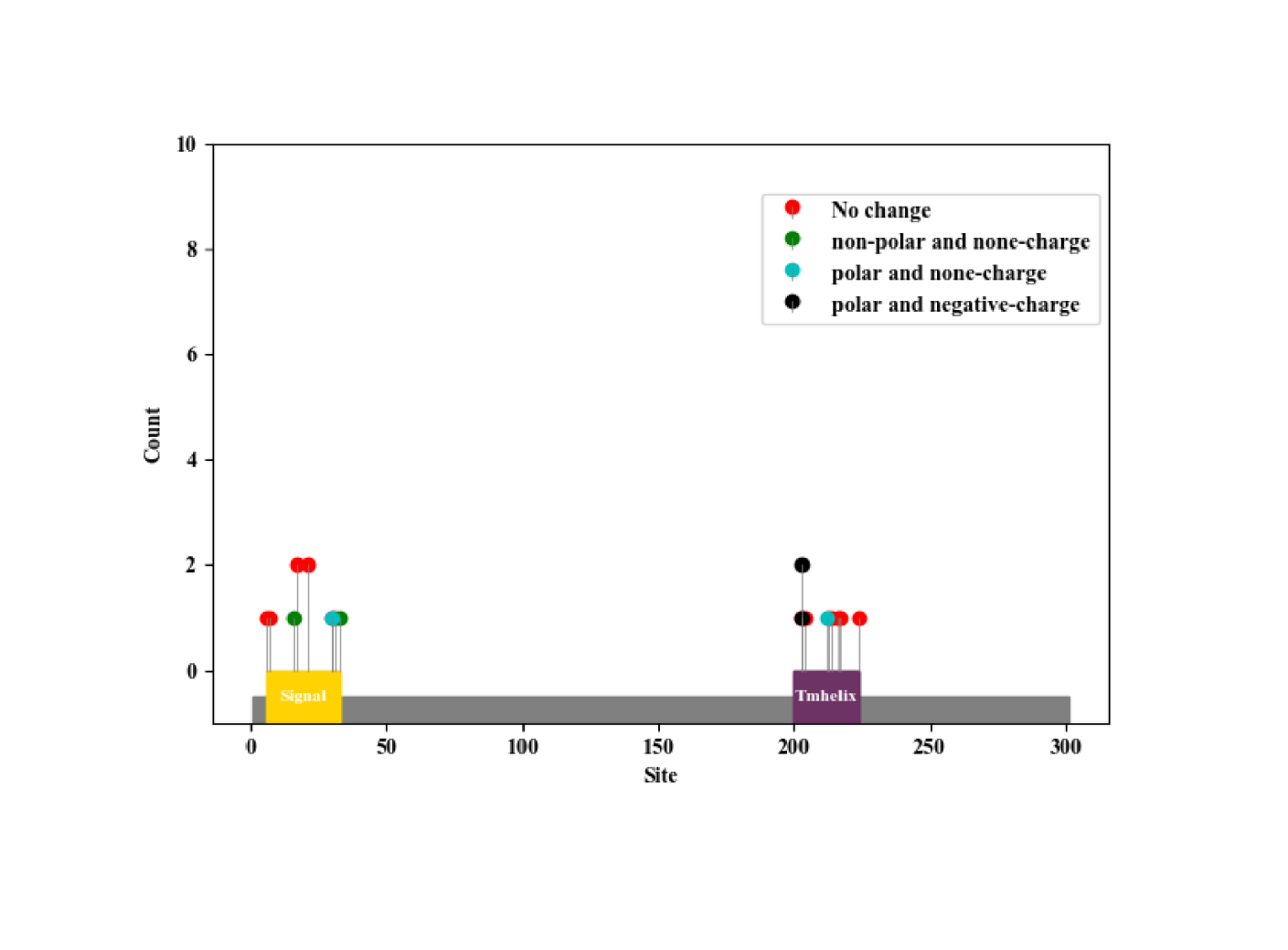


**Supplementary Figure 4.** Amino acid mutation analysis of *Ginkgo biloba* picorna-like virus. Lollipop plot depicting the location and frequency of amino acid mutations identified of the signal peptide and transmembrane helix of *Ginkgo biloba* picorna-like virus. The X-axis represents the amino acid positions, the Y-axis shows the total number of mutations, and the color indicates the type of mutation.

Figure S5


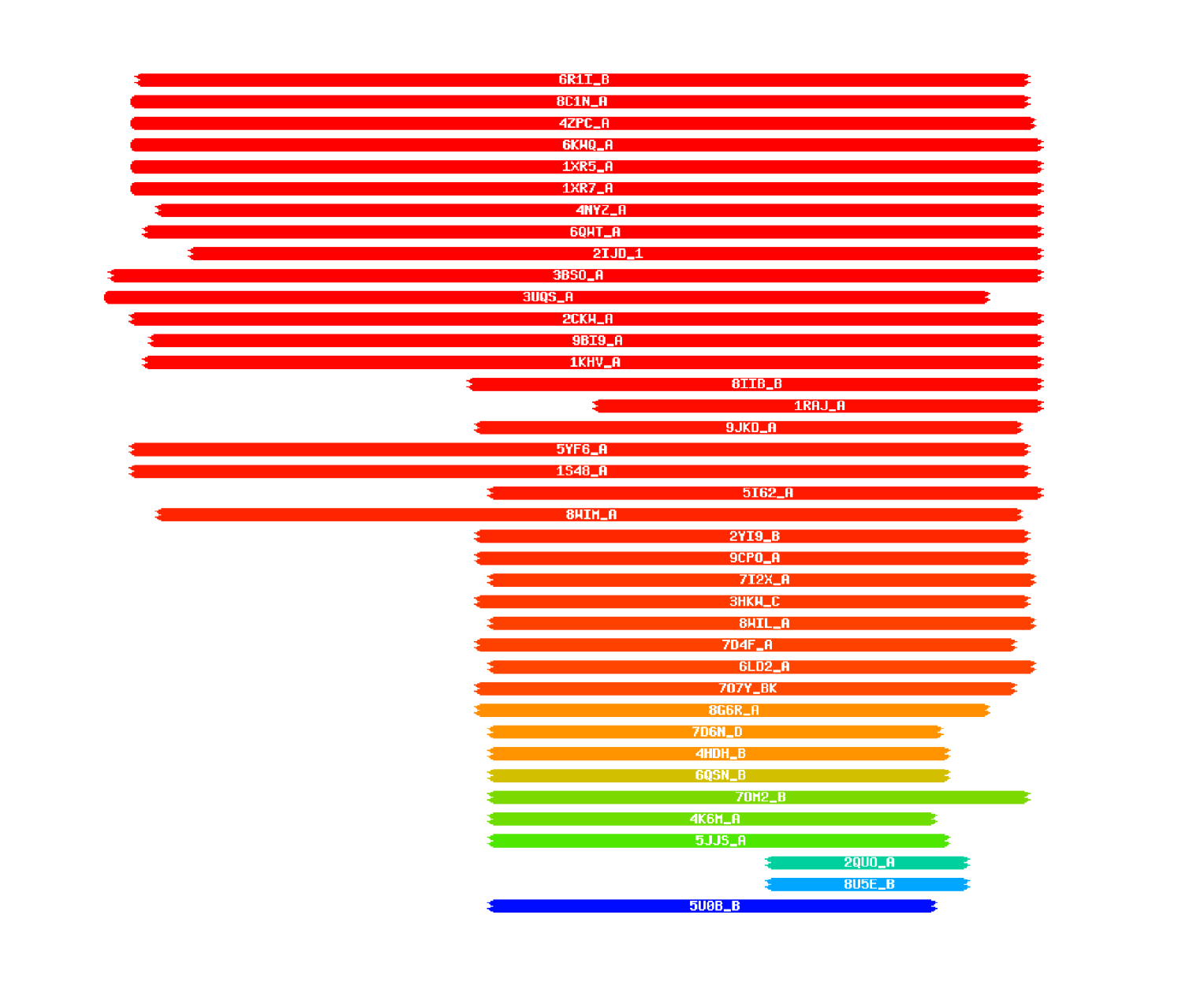


**Supplementary Figure 5.** The amino acid sequence of *Ginkgo biloba* dicistrovirus was compared with that in the HHpred database. During the comparison process, HHpred would calculate the similarity score between the two HMM models and sort the matching results based on the score. From top to bottom, the scores decrease in turn.

Figure S6


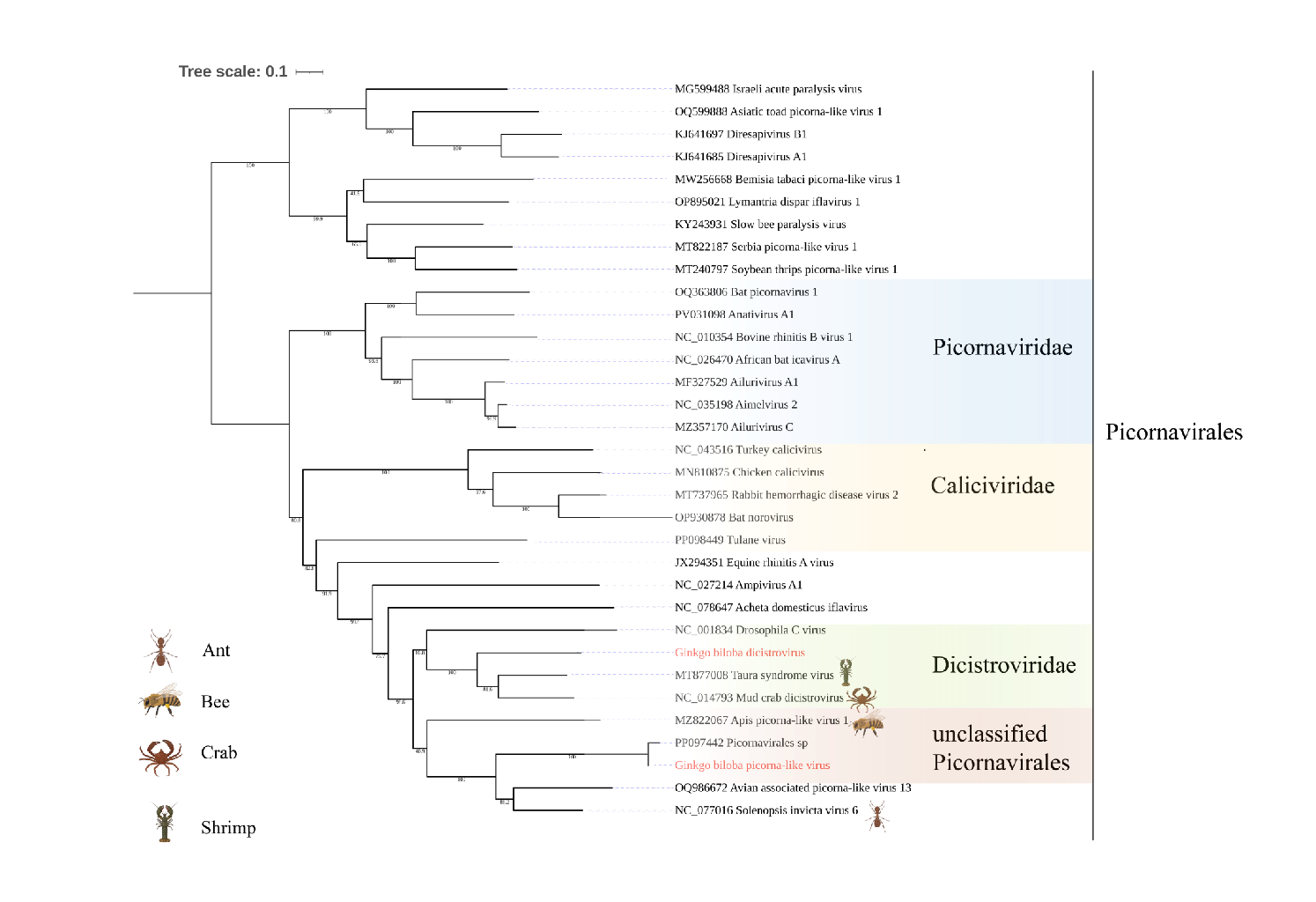


Supplementary Figure 6. Phylogenetic analysis of *Ginkgo biloba* picorna-like virus and *Ginkgo biloba* dicistrovirus. The evolutionary tree constructed by *Ginkgo biloba* picorna-like virus and *Ginkgo biloba* dicistrovirus showed that the discovered viruses are displayed in red.
